# Two Homely Remedies

**Published:** 1909-07-31

**Authors:** 


### TWO HOMELY REMEDIES.

Hedge Mustard for Laryngitis.—The common hedge mustard, *Sisymbrium officinale*, was official in the codex of 1884 under the name of Erysimum, but it has since been discarded. Saintignow has recently revived some interest in it, and it is stated that the plant is of real value in the treatment of inflammatory affections of the pharyngeal or laryngeal mucous membranes. An infusion of the leaves taken as such, or as syrup, will rapidly cure an acute attack of laryngitis often in four and twenty hours. It is specially valuable in simple laryngitis, whether acute or chronic.

Mucilage of Leeks for similiar conditions is a remedy that was recorded by Aristotle, but it has been forgotten except by a few. The mucilage is prepared by boiling leeks until the acrid volatile oil has been expelled, and then evaporating the expressed decoction to the consistence of a mucilage. The latter has an emollient action which is exceedingly grateful in cases of smoker's throat and pharyngitis, whether acute or chronic. It is also valuable in conditions of catarrh in the bigger bronchial tubes.

